# A New Method Based on Locally Optimal Step Length in Accelerated Gradient Descent for Quantum State Tomography

**DOI:** 10.3390/s24175464

**Published:** 2024-08-23

**Authors:** Mohammad Dolatabadi, Vincenzo Loia, Pierluigi Siano

**Affiliations:** Department of Management & Innovation Systems, University of Salerno, Via Giovanni Paolo II, 132, 84084 Fisciano, SA, Italy; mh.dolatabadi@gmail.com (M.D.); psiano@unisa.it (P.S.)

**Keywords:** quantum state tomography, accelerated gradient descent, non-convex optimization, positive operator-valued measures

## Abstract

Quantum state tomography (QST) is one of the key steps in determining the state of the quantum system, which is essential for understanding and controlling it. With statistical data from measurements and Positive Operator-Valued Measures (POVMs), the goal of QST is to find a density operator that best fits the measurement data. Several optimization-based methods have been proposed for QST, and one of the most successful approaches is based on Accelerated Gradient Descent (AGD) with fixed step length. While AGD with fixed step size is easy to implement, it is computationally inefficient when the computational time required to calculate the gradient is high. In this paper, we propose a new optimal method for step-length adaptation, which results in a much faster version of AGD for QST. Numerical results confirm that the proposed method is much more time-efficient than other similar methods due to the optimized step size.

## 1. Introduction

Quantum physics emerged from Albert Einstein’s efforts to explain the “Photoelectric effect”, which suggested that light can behave like a particle. Other scientists explored the alternative idea that particles such as electrons can behave like waves [1], and this wave-like behavior of particles was later mathematically formulated by Erwin Schrödinger. Schrödinger’s equation provides a theoretical foundation for quantum mechanics, but when it comes to making measurements and interpreting experimental data, statistical tools are essential. One of these tools is quantum state tomography (QST), which is briefly explained in the next section.

In quantum computing and quantum information theory, QST is one crucial step in determining the state of a quantum system, which is essential for understanding and controlling a given quantum system. It uses many identical particles, each measured in a slightly different way. By piecing together these measurements, scientists can build a picture of the original quantum state. In QST, projections are the results of measurements on the quantum system, expressed as probabilities and expectation values.

It is worth noting that QST is a general concept that can be applied to any quantum system, including digital quantum computers [2] and analog quantum simulators (computers) [3]. In [3], a tomography approach (described in terms of Positive Operator-Valued Measures (POVMs) formalism) that is implementable on analog quantum simulators, including ultra-cold atoms, is proposed. Additionally, since quantum sensing and imaging technologies have a lot of exciting applications in optical measurements, using entanglement with applications in quantum lithography [4], one of the applications of QST is in quantum sensing [5].

The QST problem can be formulated as a smooth optimization problem, and as a result, gradient-based methods [6] can be used to solve the problem. If we denote the value of the objective function of the optimization (minimization) problem at point x by f(x), the gradient descent uses $x_{k+1}=x_{k}-\varepsilon \nabla f(x_{k})$, where $\nabla f(x_{k})$ is the gradient of f at $x_{k}$, which is a vector consisting of the first-order partial derivatives of f with respect to the decision variables, and ε is a small number and represents the step size. However, while gradient-based methods are easy to implement and highly scalable, they are very slow to converge. 

Another approach is to generalize the iteration procedure by using $x_{k+1}=x_{k}-\varepsilon D\nabla f(x_{k})$, where D is a square matrix. Note that if we set D to be equal to the identity matrix then we will arrive at $x_{k+1}=x_{k}-\varepsilon \nabla f(x)$, as in gradient descent. 

If we set $D={\left(\nabla^{2}f\left(x\right)\right)}^{-1}$, then we will have the Newton method [6], which is very fast to converge in terms of the number of iterations, but ${\left(\nabla^{2}f\left(x\right)\right)}^{-1}$ is not only challenging and time-consuming to compute, but also requires a lot of memory (RAM). Now, one solution is to use less idealistic but more practical choices of D. For instance, L-BFGS [7] tries to directly approximate the vector ${\left(\nabla^{2}f\left(x\right)\right)}^{-1}\nabla f(x)$ using a limited number of previous gradients stored in RAM. However, another class of methods that are less demanding in terms of memory (RAM) usage are Accelerated Gradient Descent (AGD) methods [8,9,10], which are based on running two iterative procedures simultaneously. The advantages of this method are its ease of implementation, relatively fast convergence, and being less memory-intensive compared to L-BFGS.

To solve the QST problem, a solver based on Accelerated Gradient Descent (AGD) followed by the singular value decomposition (SVD) projection step is proposed in [2]. A MATLAB implementation is available on GitHub [11]. In [12], compressed sensing (CS) is proposed for QST. In [13], AGD is applied again; SVD projection is bypassed by introducing a non-convex programming formulation of the QST problem, and the Python code is made available [14]. A neural network-based method is presented in [15], and the code is available on GitHub [16]. In [17], a combination of CS and projected least squares (PLS) is proposed, and the code is available in [18]. In [19], attention mechanisms are used in neural networks on informationally complete POVMs. For digital quantum computers, the complexity of tomography scales exponentially with the number of qubits. To address the challenge of exponential scaling, in [20], POVMs are approximated with low-rank approximation (rank-1 projectors of the K = 6 eigenstates of the Pauli matrices), which is similar to the approach that we use for generating simulated data to validate the proposed method. The authors in [5] performed QST on pure tomography sensing using singular value decomposition (SVD) techniques for a 2-qubit system to come up with a robust method.

In this paper, we propose a new method based on the modification of AGD for a non-convex programming formulation. In our approach, one of two step sizes is adaptively chosen which increases the speed of convergence, as demonstrated in the numerical evaluations carried out. A comparison of similarities and differences between the proposed method and the top two most similar methods is presented in Table 1. The proposed method combines Momentum-inspired Factorized Gradient Descent (MiFGD) with an adaptive step length. The novel contribution of the proposed method is the introduction of a closed-form solution for locally optimal step length in AGD, significantly enhancing the convergence speed of the original MiFGD.

## 2. Introduction to Quantum Tomography

Quantum physics began with Albert Einstein’s attempts to explain the “Photoelectric effect”. Einstein’s theory suggested that the energy carried by each single energy packet (quantum) of light can be computed as follows:

(1) E=hf

where f is the frequency of the light and h is Planck’s constant. In 1924, Louis de Broglie suggested that particles such as electrons can behave like waves [1], and the equation for such wavy behavior was then proposed by Erwin Schrödinger:

(2) $$ih\frac{\partial \Psi (x,t)}{\partial t}=\left[-\frac{h^{2}}{2m}\frac{\partial^{2}}{\partial x^{2}}+V(x,t)\right]\Psi (x,t)$$

for matter particles with mass m, where V(x,t) is the potential. Indeed, physical quantities that physicists are interested in measuring, such as position, energy, momentum, and spin, are represented by Hermitian operators (acting on a Hilbert space), which are called observables. Since these operators are Hermitian, their eigenvalues are real, corresponding to the possible measurement outcomes of their corresponding observable. For example, the eigenvalues of the Hamiltonian operator $\hat{H}=\left[-\frac{h^{2}}{2m}\frac{\partial^{2}}{\partial x^{2}}+V(x,t)\right]$ are real numbers that correspond to the energy of the particle.

Indeed, it can be shown that if $E_{n}$ is an eigenvalue with the corresponding eigenfunction $\psi_{E_{n}}\left(x\right)$, which is to say that $\hat{H}\psi_{E_{n}}\left(x\right)=E_{n}\psi_{E_{n}}\left(x\right)$, then $\Psi_{n}\left(x,t\right)=e^{-iE_{n}t/h}\psi_{E_{n}}\left(x\right)$ is a solution for (2), which is called a wave function.

It can be shown that with some assumptions in Ket–Bra notation, we have the following:

(3) $$\langle \Psi_{n}\left(x,t\right){|\Psi }_{m}\left(x,t\right)\rangle =0,\;m\neq n$$

(4) $$\langle \Psi_{m}\left(x,t\right){|\Psi }_{m}\left(x,t\right)\rangle =1$$

Also, the general solution of (2) is the following:

(5) $$\Psi \left(x,t\right)=\sum_{n=0}^{\infty }C_{n}\Psi_{n}(x,t)$$

where $C_{1},C_{2},C_{3},\ldots .$ are complex numbers.

Now, it is easy to see that

(6) $$\langle \Psi \left(x,t\right)|\Psi \left(x,t\right)\rangle ={\left|C_{1}\right|}^{2}+{\left|C_{2}\right|}^{2}+{\left|C_{3}\right|}^{2}+\ldots$$

It is assumed that ${\left|C_{1}\right|}^{2}+{\left|C_{2}\right|}^{2}+{\left|C_{3}\right|}^{2}+\ldots =1$.

Max Born (1882–1970) suggested that the set of possible outcomes is exclusively restricted to $\Psi_{1}\left(x,t\right),\Psi_{2}\left(x,t\right),\Psi_{3}\left(x,t\right),\ldots$ but with different probabilities. These probabilities are proportional to ${\left|C_{1}\right|}^{2},{\left|C_{2}\right|}^{2}$, ${\left|C_{3}\right|}^{2}$, ….

Now, if we define $P_{m}=\left|\Psi_{m}\times \Psi_{m}\right|$, then it is easy to see that

(7) $$P_{m}\left.|\Psi \;\right\rangle =C_{m}\left.|\Psi_{m}\right\rangle$$

Equation (7) means that $P_{m}$ projects $\left.|\Psi \right\rangle$ onto $\left.|\Psi_{m}\right\rangle$, and for this reason $P_{m}$ is called a projector.

Another property which immediately follows from (5) and (7) is the following:

(8) $$\left(\sum_{n=0}^{\infty }P_{n}\right)\left.|\Psi \right\rangle =\sum_{n=0}^{\infty }P_{n}\left.|\Psi \right\rangle =\sum_{n=0}^{\infty }C_{n}\left.|\Psi_{n}\right\rangle =\left.|\Psi \right\rangle$$

which says that $\sum_{n=0}^{\infty }P_{n}=I$, where *I* is the identity operator.

Also, note that $P_{n}$ is a positive semi-definite (PSD) matrix.

Now, if we define $\varrho =\left|\Psi \times \Psi \right|$, then it is obvious that ϱ is PSD and

(9) $$\varrho =\left|\Psi \times \Psi \right|=\left(\sum_{n=0}^{\infty }C_{n}\left.|\Psi_{n}\;\right\rangle \right)\left(\sum_{m=0}^{\infty }C_{m}^{*}\left\langle \Psi_{m}\right.|\right)$$

As a result, $\varrho_{ij}=C_{i}C_{j}^{*}$, and therefore $trace(\varrho ){=\left|C_{1}\right|}^{2}$ + ${\left|C_{2}\right|}^{2}$ + ${\left|C_{3}\right|}^{2}$ + … = 1.

Now, if $y_{m}$ denotes the probability of outcome m, then

(10) $$y_{m}=C_{m}C_{m}^{*}=trace\left(\varrho P_{m}\right)$$

For this reason, ϱ is called a density operator. The projection operators $P_{1},P_{2},P_{3},\ldots$ can be generalized to PSD matrices $M_{1},M_{2},M_{3},\ldots$, such that $\sum_{n=0}^{\infty }M_{n}=I$. In this case, they are referred to as POVMs.

Now, given the statistical data $y_{1},\ldots ,y_{m}$ and the POVMs $M_{1},M_{2},M_{3},\ldots$, the goal is to find a PSD density operator ϱ that best fits the measurement data $y_{1},\ldots ,y_{m}$.

In other words, we want to solve the following optimization problem:

(11) $$\min\sum_{i}{\left\|y_{i}-trace\left(\varrho M_{i}\right)\right\|}^{2}\;\;s.t\;trace\left(\varrho \right)=1\;\&\;\varrho \;is\;PSD$$

Optimization (11) is called quantum state tomography (QST) [2]. In quantum computing and quantum information theory, quantum state tomography (QST) is one of the key steps in determining the state of the quantum system, which is crucial as this is key to understanding and controlling the quantum system. The term “tomography” comes from the ancient Greek words “tomos” (“slice” or “section”) and “grapho” (“to draw” or “to write”) [21]. Regular X-ray tomography (such as CT scans in medicine) performs a 3D reconstruction of an object (which cannot be directly seen) by piecing together 2D X-ray images (measurements) taken from different angles or sections to build up a 3D picture of the original unknown object in the body. QST, to a large extent, is similar, but instead of 2D X-ray images (2D X-ray projections) taken from different angles or perspectives, QST uses multiple measurements on many identical copies of the same quantum system, each measured in a slightly different way (by projecting the state onto various bases). The outcomes of these measurements are gathered as statistical data. Again, like X-ray tomography, in which complex algorithms are used to process 2D X-ray data to reconstruct a 3D image in QST, mathematical optimization based on linear algebra, like (11), is used to reconstruct the density matrix ϱ (which fully describes the quantum state). Having gathered the statistical data $y_{1},\ldots ,y_{m}$, what these optimization techniques (for instance, least squares fitting) do is to fit a model (the density matrix ϱ) that best fits the measurement data $y_{1},\ldots ,y_{m}$. Here, we must deal with statistical noise from quantum measurements and reconstruct the most probable quantum state, despite measurement imperfections. The main difference here is that while 3D reconstruction in X-ray tomography can be undertaken via a series of measurements on the same (classical) object, in the case of a single quantum particle, measurement perturbs the state of the quantum particle, often making its further investigation uninformative [21]. We use a source that creates many identical particles in the same unknown quantum state. So, tomography uses many identical particles, each measured in a slightly different way. By piecing together these measurements, scientists can build a picture of the original quantum state.

## 3. Proposed Adaptive Method for QST

As problem (11) does not have a closed-form solution, we need to use an iterative approach to solve it.

In [13], problem (11) is formulated as follows:

(12) $$min\sum_{i}{\left\|y_{i}-trace\left(UU^{†}M_{i}\right)\right\|}^{2}\;\;s.t\;{\left\|U\right\|}^{2}=1,\;U\in C^{d\times r}$$

in which the matrix U is assumed to be low rank. Also, $\left\|U\right\|$ is the Frobenius norm of U, which means that

(13) $${\left\|U\right\|}^{2}=\sum_{j}\sum_{i}{\left\|U_{ij}\right\|}^{2}=\sum_{j}\sum_{i}U_{ij}U_{ij}^{*}\;\;\;\;$$

Then, an Accelerated Gradient Descent (AGD) method called MiFGD is proposed [8], as follows:

(14) $$U_{k+1}=Z_{k}-\eta \left[\sum_{i}\left(trace\left(Z_{k}Z_{k}^{†}M_{i}\right)-y_{i}\right)M_{i}\right]Z_{k}$$

(15) $$Z_{k+1}=U_{k+1}+\mu (U_{k+1}-U_{k})$$

in which η and μ are two fixed step sizes.

**Figure 1:**
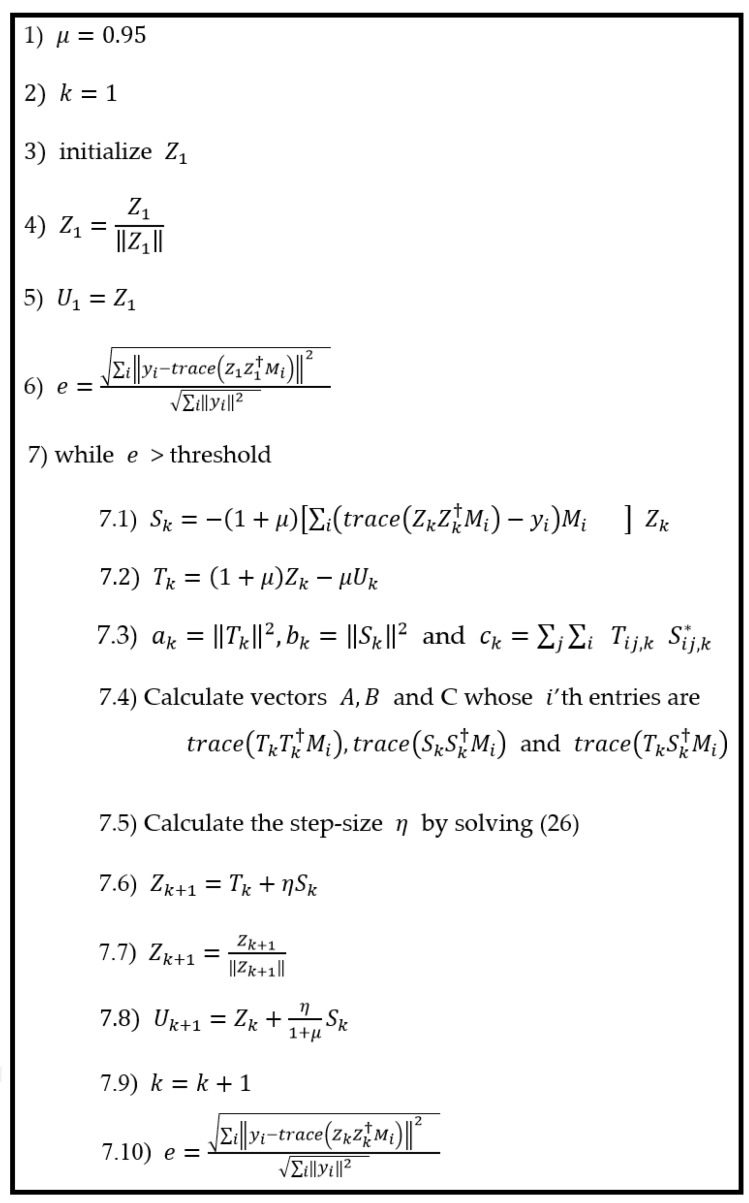
The proposed method: AGD with optimal step length η.

In this paper, we present a method for finding a closed-form solution for the greedy optimal choice of η, which means choosing η in a way that results in the biggest decrease in the objective function in that iteration. A description of the proposed method is given below and in Figure 1.

Here, it is worth mentioning that we keep the other step size μ constant, which is set to μ=0.95 inourexperiments.

For this reason, in two consecutive steps, there is a possibility that while η is chosen to result in a lower error in the next iteration, the step size μ will remain out of our control and may push the iterative approach away from what we hope to see for the next run. However, the method still performs much better than MiFGD overall. This is something that we will verify in our experiments (see Section 4).

Let us start with rewriting (15) as follows:

(16) $$Z_{k+1}=U_{k+1}+\mu \left(U_{k+1}-U_{k}\right)=\left(1+\mu \right)U_{k+1}-{\mu U}_{k}$$

Now, if we plug in $U_{k+1}$ from (14), we arrive at the following:

(17) $$Z_{k+1}=T_{k}+\eta S_{k}$$

in which

(18) $$S_{k}=-\left(1+\mu \right)\left[\sum_{i}\left(trace\left(Z_{k}Z_{k}^{†}M_{i}\right)-y_{i}\right)M_{i}\right]Z_{k}$$

and

(19) $$T_{k}=\left(1+\mu \right)Z_{k}-{\mu U}_{k}$$

As we want $Z_{k+1}$ to be normalized, we need to minimize

$$\sum_{i}{\left\|y_{i}-trace\left(\frac{Z_{k+1}}{\left\|Z_{k+1}\right\|}\frac{Z_{k+1}^{†}}{\left\|Z_{k+1}\right\|}M_{i}\right)\right\|}^{2}$$

We consider that

(20) $$argmin\sum_{i}{\left\|y_{i}-trace\left(\frac{Z_{k+1}}{\left\|Z_{k+1}\right\|}\frac{Z_{k+1}^{†}}{\left\|Z_{k+1}\right\|}M_{i}\right)\right\|}^{2}=argmin\sum_{i}{\left\|y_{i}{\left\|Z_{k+1}\right\|}^{2}-trace\left(Z_{k+1}Z_{k+1}^{†}M_{i}\right)\right\|}^{2}\;\;$$

It can be obtained that

(21) $${\left\|Z_{k+1}\right\|}^{2}={\left\|T_{k}\right\|}^{2}+\eta^{2}{\left\|S_{k}\right\|}^{2}+2\eta \sum_{j}\sum_{i}\;T_{ij,k}\;S_{ij,k}^{*}$$

Also, note that ${\left\|T_{k}\right\|}^{2},{\left\|S_{k}\right\|}^{2}$ and $\sum_{j}\sum_{i}T_{ij,k}S_{ij,k}^{*}$ are real numbers independent of η; therefore, if we set ${a_{k}=\left\|T_{k}\right\|}^{2},b_{k}={\left\|S_{k}\right\|}^{2}$, and $c_{k}=\sum_{j}\sum_{i}T_{ij,k}S_{ij,k}^{*}$, then

(22) $${\left\|Z_{k+1}\right\|}^{2}=a_{k}+{b_{k}\eta }^{2}+{2c}_{k}\eta$$

Similarly, we have

(23) $$trace\left(Z_{k+1}Z_{k+1}^{†}M_{i}\right)=trace\left(T_{k}T_{k}^{†}M_{i}\right)+\eta^{2}trace\left(S_{k}S_{k}^{†}M_{i}\right)+2\eta trace\left(T_{k}S_{k}^{†}M_{i}\right)$$

Now, if we define vectors A,B,C and Y to be vectors whose i’th entries are $trace\left(T_{k}T_{k}^{†}M_{i}\right),trace\left(S_{k}S_{k}^{†}M_{i}\right),trace\left(T_{k}S_{k}^{†}M_{i}\right)$ and $y_{i}$, respectively, then from (20) and (23), we have

(24) $$\begin{array}{cc} \; & argmin\sum_{i}{∥y_{i}-trace\left(\frac{Z_{k+1}}{∥Z_{k+1}∥}\frac{Z_{k+1}^{†}}{∥z_{k+1}∥}M_{i}\right)∥}^{2}=argmin∥Y∥Z_{k+1}{∥}^{2}-A-\eta^{2}B-2\eta C{∥}^{2}=\\ \; & argmin{\left(Y{∥Z_{k+1}∥}^{2}-A-\eta^{2}B-2\eta C\right)}^{T}\left(Y{∥Z_{k+1}∥}^{2}-A-\eta^{2}B-2\eta C\right)=Y^{T}Y{∥Z_{k+1}∥}^{4}-\\ \; & 2Y^{T}A{∥Z_{k+1}∥}^{2}-2\eta^{2}Y^{T}B{∥Z_{k+1}∥}^{2}-4\eta Y^{T}C{∥Z_{k+1}∥}^{2}+A^{T}A+2\eta^{2}A^{T}B+4\eta A^{T}C+\\ \; & \eta^{4}B^{T}B+4\eta^{3}B^{T}C+4\eta^{2}C^{T}C \end{array}$$

It is worth noting that while A,B,C and Y are vectors, ${Y^{T}Y,Y}^{T}A,Y^{T}B,Y^{T}C,A^{T}A$ $,A^{T}B,A^{T}C,B^{T}B,B^{T}C$ and $C^{T}C$ are all real numbers independent of η.

Now, from (22) and (24), we can conclude that we have to minimize the following 4th-order polynomial with respect to η:

(25) $$\begin{array}{cc} \; & Y^{T}Ya^{2}-2Y^{T}Aa+A^{T}A+\eta \left(4Y^{T}Yac-4Y^{T}Ca-4Y^{T}Ac+4A^{T}C\right)+\eta^{2}\left(4C^{T}C+2A^{T}B-\right.\\ \; & \left.2Y^{T}Ba-2Y^{T}Ab-8Y^{T}Cc+4Y^{T}Yc^{2}+2Y^{T}Yab\right)+\eta^{3}\left(4Y^{T}Ybc-4Y^{T}Cb-4Y^{T}Bc+\right.\\ \; & \left.4B^{T}C\right)+\eta^{4}\left(Y^{T}Yb^{2}-2Y^{T}Bb+B^{T}B\right) \end{array}$$

This attains a minimum where the derivative with respect to η is zero:

(26) $$\begin{array}{cc} \; & \left(4Y^{T}Yac-4Y^{T}Ca-4Y^{T}Ac+4A^{T}C\right)+\eta \left(8C^{T}C+4A^{T}B-4Y^{T}Ba-4Y^{T}Ab-16Y^{T}Cc+\right.\\ \; & \left.8Y^{T}Yc^{2}+4Y^{T}Yab\right)+\eta^{2}\left(12Y^{T}Ybc-12Y^{T}Cb-12Y^{T}Bc+12B^{T}C\right)+\eta^{3}\left(4Y^{T}Yb^{2}-8Y^{T}Bb+\right.\\ \; & \left.4B^{T}B\right)=0 \end{array}$$

Now, by solving (26), which is a humble cubic equation with respect to η, the optimal step size for the kth step is obtained.

Note that computing A, B, and C at each step is the most time-consuming operation, which makes the proposed method almost three times slower at each iteration compared to MiFGD, but as we choose a much more optimized step size, our approach becomes faster overall. The numerical results are presented in Section 5.

## 4. Numerical Results

While what we have explained in the previous section applies to general quantum systems, in this section, we apply it to multi-qubit spin-half systems.

Suppose that we have a particle that only has two states, and the state of the particle has two entries:

(27) $$\left(\begin{array}{c} \psi \left(x_{1}\right)\\ \psi \left(x_{2}\right) \end{array}\right)=\left(\;\begin{array}{c} \alpha \\ \beta \end{array}\right)$$

where α and β are two complex numbers and ${\left|\alpha \right|}^{2}$ and ${\left|\beta \right|}^{2}$ are the probabilities of being at each of the two states. The discretization of the Hamiltonian is a 2 by 2 matrix. On the other hand, since the Hamiltonian as an operator is expected to be Hermitian, which guarantees that the eigenvalues are real (and as a result, they correspond to the possible measurement outcomes of their corresponding observable), physicists investigate the space of all possible 2 by 2 Hamiltonians that satisfy the following equations:

(28) $$H_{11}^{*}=H_{11}$$

(29) $$H_{12}^{*}=H_{21}$$

(30) $$H_{22}^{*}=H_{22}$$

As a result, both $H_{11}$ and $H_{22}$ should be real numbers, and $H_{21}$ should be the conjugate of $H_{12}$. After straightforward calculation, physicists arrive at the following as the general formula for the Hamiltonian in the aforementioned binary state.

(31) $$H=\frac{1}{2}\left(I+r_{x}\sigma_{1}+r_{y}\sigma_{2}+r_{z}\sigma_{3}\right)\;$$

in which

(32) $$\sigma_{1}=\left(\begin{array}{cc} 0 & 1\\ 1 & 0 \end{array}\right),\sigma_{2}=\left(\begin{array}{cc} 0 & -i\\ i & 0 \end{array}\right),\sigma_{3}=\left(\begin{array}{cc} 1 & 0\\ 0 & -1 \end{array}\right)$$

which are called Pauli matrices.

Additionally, the following matrices can be defined:

(33) $$S_{x}=\frac{h}{2}\sigma_{1},S_{y}=\frac{h}{2}\sigma_{2},S_{z}=\frac{h}{2}\sigma_{3}$$

These satisfy the following properties:

(34) $$S_{x}S_{y}-S_{y}S_{x}=ihS_{z}$$

(35) $$S_{y}S_{z}-S_{z}S_{y}=ihS_{x}$$

(36) $$S_{z}S_{x}-S_{x}S_{z}=ihS_{y}$$

The above three equalities remind physicists of angular momentum. For this reason, they relate them to the spin system. They call the system “spin-1/2” because of the coefficient $\frac{h}{2}$ (in Equation (33)), which is the component of angular momentum. These spin-1/2 systems are interesting since they have two states and are the building blocks for qubits (in digital quantum computers).

Now, if we assume that, for example, the density operator in the case of a single qubit is

(37) $$\varrho =\frac{1}{2}\left(I+r_{x}\sigma_{1}+r_{y}\sigma_{2}+r_{z}\sigma_{3}\right)$$

then

(38) $$trace\left(\varrho \sigma_{1}\right)=\frac{1}{2}trace\left(\sigma_{1}+r_{x}\sigma_{1}\sigma_{1}+r_{y}\sigma_{2}\sigma_{1}+r_{z}\sigma_{3}\sigma_{1}\right)=\frac{1}{2}trace\left(r_{x}I\right)=r_{x}$$

and from (10) we can calculate $r_{x}$ from our measurement data. Similarly, we can calculate from our measurement data the other coefficients, $r_{y}$ and $r_{z}$, which allows us to identify the density matrix ϱ. This is the simplest form of QST. But for more complicated cases (multi-qubit systems), more has to be undertaken.

It is easy to see that

(39) $$\sigma_{3}\left[\begin{array}{c} 1\\ 0 \end{array}\right]=\frac{h}{2}\left[\begin{array}{c} 1\\ 0 \end{array}\right]$$

(40) $$\sigma_{3}\left[\begin{array}{c} 0\\ 1 \end{array}\right]=-\frac{h}{2}\left[\begin{array}{c} 0\\ 1 \end{array}\right]$$

Here, $\left[\begin{array}{c} 1\\ 0 \end{array}\right]$ is called spin-up and is denoted by $\left.|↑\right\rangle$. Also, $\left[\begin{array}{c} 0\\ 1 \end{array}\right]$ is called spin down and is denoted by $\left.|↓\right\rangle$.

Therefore, Equations (39) and (40) are sometimes expressed as follows:

(41) $$\sigma_{3}\left.|↑\right\rangle =\frac{h}{2}\left.|↑\right\rangle$$

(42) $$\sigma_{3}\left.|↓\right\rangle =-\frac{h}{2}\left.|↓\right\rangle$$

which indicates that the eigenstates of $\sigma_{3}$ are $\left.|↑\right\rangle$ and $\left.|↓\right\rangle$, with the corresponding eigenvalues $\frac{h}{2}$ and $-\frac{h}{2}$, respectively. Also, it can be seen that the following two vectors are the eigenvectors for $\sigma_{1}$:

(43) $$\left.|x,↑\right\rangle =\frac{1}{\sqrt{2}}\left(\left.|↑\right\rangle +\left.|↓\right\rangle \right)$$

(44) $$\left.|x,↓\right\rangle =\frac{1}{\sqrt{2}}\left(\left.|↑\right\rangle -\left.|↓\right\rangle \right)$$

and the following two are the eigenvectors for $\sigma_{2}$:

(45) $$\left.|y,↑\right\rangle =\frac{1}{\sqrt{2}}\left(\left.|↑\right\rangle +\left.i|↓\right\rangle \right)$$

(46) $$\left.|y,↓\right\rangle =\frac{1}{\sqrt{2}}\left(\left.|↑\right\rangle -i\left.|↓\right\rangle \right)$$

Furthermore, the real three-dimensional space that we live in relates to the two-dimensional complex vector space within which a qubit sits by rewriting Equation (27) as follows. Suppose that

(47) $$\psi =\alpha \left.|↑\right\rangle +\beta \;\left.|↓\right\rangle$$

where ${{\left|\alpha \right|}^{2}+\left|\beta \right|}^{2}=1$. Using polar coordinates, we have

(48) $$\psi =r_{1}e^{i\phi_{1}}\left.|↑\;\right\rangle +r_{2}e^{i\phi_{2}}\;\left.|↓\;\right\rangle =e^{i\phi_{1}}\left(r_{1}\left.|↑\;\right\rangle +r_{2}e^{i{(\phi }_{2}-\phi_{1})}\;\left.|↓\;\right\rangle \right)\;=e^{i\phi_{1}}\left(r_{1}\left.|↑\;\right\rangle +r_{2}e^{i\phi }\;\left.|↓\right\rangle \right)$$

But since $r_{1}^{2}+r_{2}^{2}=1$, we can write $r_{1}=\cos\left(\frac{\theta }{2}\right),r_{2}=\sin\left(\frac{\theta }{2}\right)$, and therefore

(49) $$\psi =e^{i\phi_{1}}\left(\cos\left(\frac{\theta }{2}\right)\left.|↑\;\right\rangle +\sin\left(\frac{\theta }{2}\right)e^{i\phi }\;\left.|↓\right\rangle \right)$$

And if we ignore $e^{i\phi_{1}}$, we end up with the following:

(50) $$\psi =\cos\left(\frac{\theta }{2}\right)\left.|↑\;\right\rangle +\sin\left(\frac{\theta }{2}\right)e^{i\phi }\left.|↓\right\rangle$$

which is called the Bloch sphere representation of the quantum state.

The Bloch sphere representation (50) is important as it helps us to figure out the state of a qubit on the surface of a unit sphere in real 3D space rather than complex 2D space. Now, in this coordinate system state, “z” (when θ=0) is equivalent to $\left.|↑\right\rangle$ and “−z” (when θ=π, ϕ = 0) is equivalent to $\left.|↓\right\rangle$, which means that while in complex vector space $\left.|↑\right\rangle$ and $\left.|↓\right\rangle$ are orthogonal states, once we represent them on the Block sphere they are antipodal states that point in opposite directions. Indeed, angle θ is the angle between the unit vector representing the state of a qubit in 3D space and the axis z, and angle ϕ is the angle between the projection of the unit vector on the *x*-*y* plane and the x axis. Now, $\theta =\frac{\pi }{2}$ and ϕ=0 are equivalent to $\left.|x,↑\right\rangle =\frac{1}{\sqrt{2}}\left(\left.|↑\right\rangle +\left.|↓\right\rangle \right)$, and if $\theta =\frac{\pi }{2}$ and ϕ=π, we arrive at $\left.|x,↓\right\rangle =\frac{1}{\sqrt{2}}\left(\left.|↑\right\rangle -\left.|↓\right\rangle \right)$, and the last two vectors lie on the *x* and –*x* axes, respectively. Also, $(\theta ,\phi )=(\frac{\pi }{2}$,$\;\frac{\pi }{2})$ corresponds to $\left.|y,↑\right\rangle =\frac{1}{\sqrt{2}}\left(\left.|↑\right\rangle +\left.i|↓\right\rangle \right)$, which is in the direction of the *y* axis and, $(\theta ,\phi )=(\frac{\pi }{2}$,$-\frac{\pi }{2})$ corresponds to $\left.|y,↓\right\rangle =\frac{1}{\sqrt{2}}\left(\left.|↑\right\rangle -i\left.|↓\right\rangle \right)$, which is in the direction of the –*y* axis. Moreover, as we have

(51) $$\left.|↑\right\rangle \left\langle ↑|\right.+\left.|↓\right\rangle \left\langle ↓|\right.=\left(\begin{array}{cc} 1 & 0\\ 0 & 1 \end{array}\right)=I$$

(52) $$\left.|x,↑\right\rangle \left\langle x,↑|\right.+\left.|x,↓\right\rangle \left\langle x,↓|\right.=\left(\begin{array}{cc} 1 & 0\\ 0 & 1 \end{array}\right)=I$$

(53) $$\left.|y,↑\right\rangle \left\langle y,↑|\right.+\left.|y,↓\right\rangle \left\langle y,↓|\right.=\left(\begin{array}{cc} 1 & 0\\ 0 & 1 \end{array}\right)=I$$

in [13], the following three bases are used for QST:

(54) $$B_{1}=\left\{\left.|x,↑\right\rangle ,\left.|x,↓\right\rangle \right\}$$

(55) $$B_{2}=\left\{\left.|y,↑\right\rangle ,\left\langle y,↓|\right.\right\}$$

(56) $$B_{3}=\left\{\left.|↑\right\rangle ,\left.|↓\right\rangle \right\}$$

However, in [11], more complicated bases are introduced, as follows:

(57) $$B_{1}=\left\{W_{1}^{T},W_{2}^{T}\right\}$$

(58) $$B_{2}=\left\{W_{3}^{T},W_{4}^{T}\right\}$$

(59) $$B_{3}=\left\{W_{5}^{T},W_{6}^{T}\right\}$$

where $W_{1},\ldots ,W_{6}$ are the rows of the following matrix:

(60) $$W=\left[\begin{array}{cc} \cos\left(\theta /2\right) & -\mathrm{isin}\left(\theta /2\right)\\ -\mathrm{isin}\left(\theta /2\right) & \cos\left(\theta /2\right)\\ \cos\left(\theta /2\right) & -\sin\left(\theta /2\right)\\ \sin\left(\theta /2\right) & \cos\left(\theta /2\right)\\ \cos\left(\theta /2\right)-\mathrm{isin}\left(\theta /2\right) & 0\\ 0 & \cos\left(\theta /2\right)+\mathrm{isin}\left(\theta /2\right) \end{array}\right]$$

Now, again, similar to (51)–(53), we have

(61) $$W_{1}^{T}W_{1}+W_{2}^{T}W_{2}=I$$

(62) $$W_{3}^{T}W_{3}+W_{4}^{T}W_{4}=I\;$$

(63) $$W_{5}^{T}W_{5}+W_{6}^{T}W_{6}=I\;$$

To obtain the above formulas, remember that, for example, what we mean by $W_{1}^{T}$ is $\left[\begin{array}{c} \cos\left(\theta /2\right)\\ \mathrm{isin}\left(\theta /2\right) \end{array}\right]$, not $\left[\begin{array}{c} \cos\left(\theta /2\right)\\ -\mathrm{isin}\left(\theta /2\right) \end{array}\right]$.

We create our data according to the procedure explained in [11]. As mentioned in [2], the reason for choosing this kind of procedure is that it allows for applications in which the measurement matrices are ill conditioned [22,23,24].

To be more specific, the proposed procedure selects (with repetitions) n rows of matrix W in $6^{n}$ different ways, and for each of these choices we compute the tensor products of the selected rows. Then, we come up with $s=6^{n}$ different row vectors, $A_{1},\ldots ,A_{s}$, each of which with $d=2^{n}$ entries. Now, for an n-qubit system, the POVMs are $M_{1}=A_{1}^{T}A_{1},M_{2}=A_{2}^{T}A_{2},\ldots ,M_{s}=A_{s}^{T}A_{s}$. Also, a random PSD matrix ϱ is created and the simulated generated data are constructed by the relation $y_{i}=trace\left(\varrho M_{i}\right)+\varepsilon_{i}$, where $\varepsilon_{i}$ is a Gaussian noise term. Now, given the POVMs $M_{1}=A_{1}^{T}A_{1},M_{2}=A_{2}^{T}A_{2},\ldots ,M_{s}=A_{s}^{T}A_{s}$ and the simulated measurement data $y_{1},\ldots ,y_{s}$, the QST algorithm should be able to find a PSD matrix ϱ such that $y_{i}\approx trace\left(\varrho M_{i}\right)$, for which we solve (12) using the proposed method. As our simulated data are constructed by $y_{i}=trace\left(\varrho M_{i}\right)+\varepsilon_{i}$, we expect that the optimal value of the problem (12) will be $\sum_{i}{\left\|\varepsilon_{i}\right\|}^{2}$, which is around 0.003 in all our experiments. So, we can approximately measure the optimality gap.

As an illustrative example of how POVMs are created, let us take n = 3. Also, suppose that $\theta =\frac{\pi }{2}$. Therefore, the matrix W is as follows:

(64) $$W=\frac{1}{\sqrt{2}}\left[\begin{array}{cc} 1 & -i\\ -i & 1\\ 1 & -1\\ 1 & 1\\ 1-i & 0\\ 0 & 1+i \end{array}\right]$$

It is easy to check that (61)–(63) are verified.

$$W_{1}^{T}W_{1}+W_{2}^{T}W_{2}=\frac{1}{\sqrt{2}}\left[\begin{array}{c} 1\\ i \end{array}\right]\times \frac{1}{\sqrt{2}}\left[\begin{array}{cc} 1 & -i \end{array}\right]+\frac{1}{\sqrt{2}}\left[\begin{array}{c} i\\ 1 \end{array}\right]\times \frac{1}{\sqrt{2}}\left[\begin{array}{cc} -i & 1 \end{array}\right]=\left[\begin{array}{cc} 1 & 0\\ 0 & 1 \end{array}\right]=I$$

$$W_{3}^{T}W_{3}+W_{4}^{T}W_{4}=\frac{1}{\sqrt{2}}\left[\begin{array}{c} 1\\ -1 \end{array}\right]\times \frac{1}{\sqrt{2}}\left[\begin{array}{cc} 1 & -1 \end{array}\right]+\frac{1}{\sqrt{2}}\left[\begin{array}{c} 1\\ 1 \end{array}\right]\times \frac{1}{\sqrt{2}}\left[\begin{array}{cc} 1 & 1 \end{array}\right]=\left[\begin{array}{cc} 1 & 0\\ 0 & 1 \end{array}\right]=I$$

$$W_{5}^{T}W_{5}+W_{6}^{T}W_{6}=\frac{1}{\sqrt{2}}\left[\begin{array}{c} 1+i\\ 0 \end{array}\right]\times \frac{1}{\sqrt{2}}\left[\begin{array}{cc} 1-i & 0 \end{array}\right]+\frac{1}{\sqrt{2}}\left[\begin{array}{c} 0\\ 1-i \end{array}\right]\times \frac{1}{\sqrt{2}}\left[\begin{array}{cc} 0 & 1+i \end{array}\right]=\left[\begin{array}{cc} 1 & 0\\ 0 & 1 \end{array}\right]=I$$

Now, we have ${s=6}^{3}$ different ways to select (with repetitions) 3 rows of matrix W, which we denote by the following 216 rows:

$$\begin{array}{ccc} 1 & 1 & 1\\ \vdots & \vdots & \vdots \\ 1 & 1 & 6 \end{array}\;\begin{array}{ccc} 1 & 2 & 1\\ \vdots & \vdots & \vdots \\ 1 & 2 & 6 \end{array}\;\begin{array}{ccc} \vdots & \vdots & \vdots \\ 1 & 6 & 1\\ \vdots & \vdots & \vdots \end{array}\;\begin{array}{ccc} 1 & 6 & 6\\ \vdots & \vdots & \vdots \\ 6 & 6 & 1 \end{array}\;\begin{array}{ccc} 6 & 6 & 2\\ \vdots & \vdots & \vdots \\ 6 & 6 & 6 \end{array}$$

Then, for example, for the 9th row, which is [1 2 3], we have to pick the corresponding rows in *W*, which are

$$W_{1}=\frac{1}{\sqrt{2}}\left[\begin{array}{cc} 1 & -i \end{array}\right]$$

$$W_{2}=\frac{1}{\sqrt{2}}\left[\begin{array}{cc} -i & 1 \end{array}\right]$$

$$W_{3}=\frac{1}{\sqrt{2}}\left[\begin{array}{cc} 1 & -1 \end{array}\right]$$

Now, as the tensor product of two row vectors is defined as

$$a⊗b=\left[a_{1}\ldots a_{n}\right]⊗\left[b_{1}\ldots b_{n}\right]=[a_{1}b_{1}\ldots a_{1}b_{n}\ldots a_{n}b_{1}\ldots a_{n}b_{n}]$$

we have

$$W_{1}⊗W_{2}=\frac{1}{2}\left[\begin{array}{cc} 1 & -i \end{array}\right]⊗\left[\begin{array}{cc} -i & 1 \end{array}\right]=\frac{1}{2}[\begin{array}{cc} -i & \;1 \end{array}-1-i]$$

and therefore

$${A_{10}=W}_{1}⊗W_{2}⊗W_{3}=\frac{1}{2\sqrt{2}}\left[\begin{array}{cc} -i & \;1 \end{array}-1-i\right]⊗\left[\begin{array}{cc} 1 & -1 \end{array}\right]\;=\frac{1}{2\sqrt{2}}[\begin{array}{cc} -i & \;i \end{array}\;1-1-1\;1-i\;i]$$

In a similar way, $A_{1},\ldots ,A_{216}$ are constructed. Now, for a 3-qubit system, the POVMs are $M_{1}=A_{1}^{T}A_{1},M_{2}=A_{2}^{T}A_{2},\ldots ,M_{216}=A_{216}^{T}A_{216}$, consisting of 216 matrices of dimensions of 8 by 8.

We apply the proposed method on a n-qubit system, where in our case n = 6, 7, 8 each with $\theta =\frac{\pi }{3}\;or\;\theta =\frac{\pi }{2}$ and rank(U) = 10 or rank(U) = d.

As a result, we will report the comparison of our method with MiFGD for 12 different configurations.

**Figure 2:**
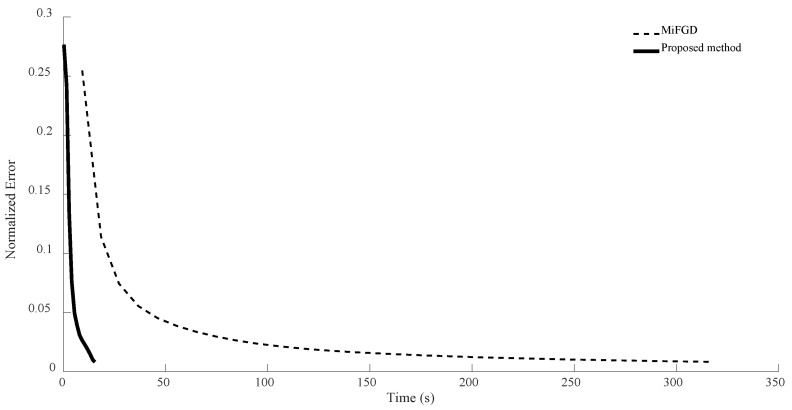
Numerical result for n = 6, $\theta =\frac{\pi }{2}$, and rank = d.

**Figure 3:**
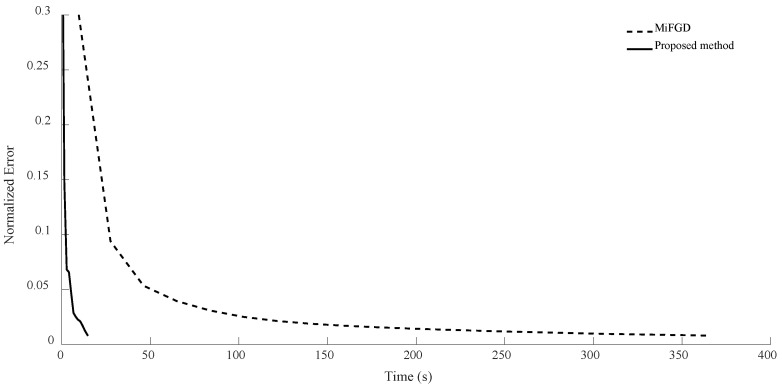
Numerical result for n = 6, $\theta =\frac{\pi }{2}$, and rank = 10.

**Figure 4:**
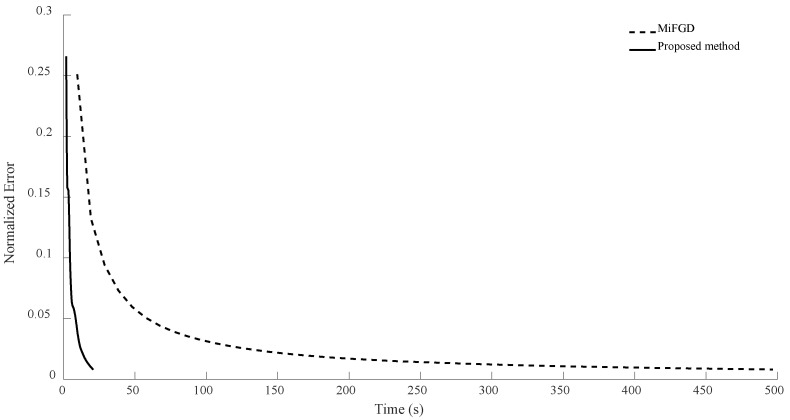
Numerical result for n = 6, $\theta =\frac{\pi }{3}$, and rank = d.

**Figure 5:**
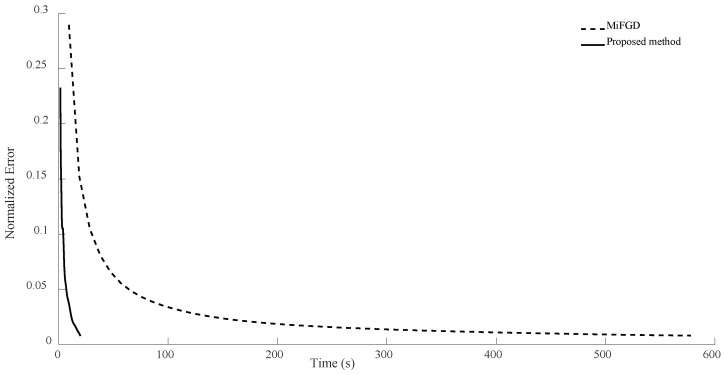
Numerical result for n = 6, $\theta =\frac{\pi }{3}$, and rank = 10.

For n = 6, we compare the performance of the two algorithms for four different configurations, $\left(\theta ,rank)=(\frac{\pi }{2},d\right)$, $\left(\theta ,rank)=(\frac{\pi }{2},10\right)$, $\left(\theta ,rank)=(\frac{\pi }{3},d\right)$, and $\left(\theta ,rank)=(\frac{\pi }{3},10\right)$, as shown in Figure 2, Figure 3, Figure 4 and Figure 5. As it can be seen, our algorithm reaches the same accuracy of MiFGD approximately 25 times faster.

To be more specific, for instance, in the first configuration (as seen in Figure 2), after 3480 iterations and spending 318 s, MiFGD reaches a normalized error of 0.0082, while our method after just 61 iterations in 15 s reaches a normalized error of 0.0080.

As can be seen, the proposed method takes 0.24 s per iteration, while MiFGD takes 0.09 s, which means that the proposed method is 2.7 times slower per iteration, but overall much faster thanks to a very well-optimized step length.

A summary of the results is presented in Table 2. As it can be seen, although the elapsed time per iteration when using the proposed method is higher due to the fact that the number of expensive operations (computing $trace\left(T_{k}T_{k}^{†}M_{i}\right),trace\left(S_{k}S_{k}^{†}M_{i}\right)$ and $trace\left(T_{k}S_{k}^{†}M_{i}\right)$) is tripled compared to MiFGD (in which the only expensive operation is the calculation of $trace\left(Z_{k}Z_{k}^{†}M_{i}\right)$), both the number of iterations and the elapsed time have decreased in the proposed method.

**Figure 6:**
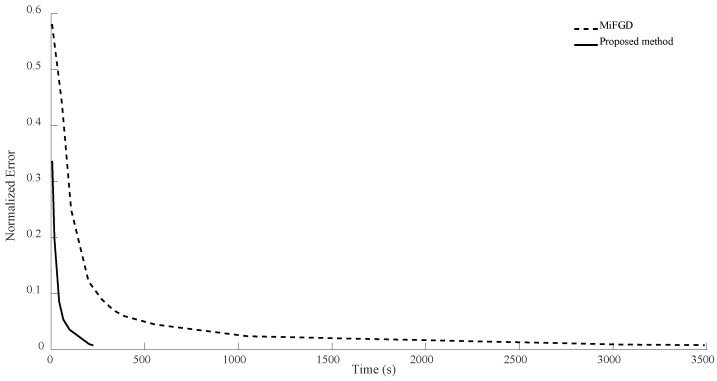
Numerical result for n = 7, $\theta =\frac{\pi }{2}$, and rank = d.

**Figure 7:**
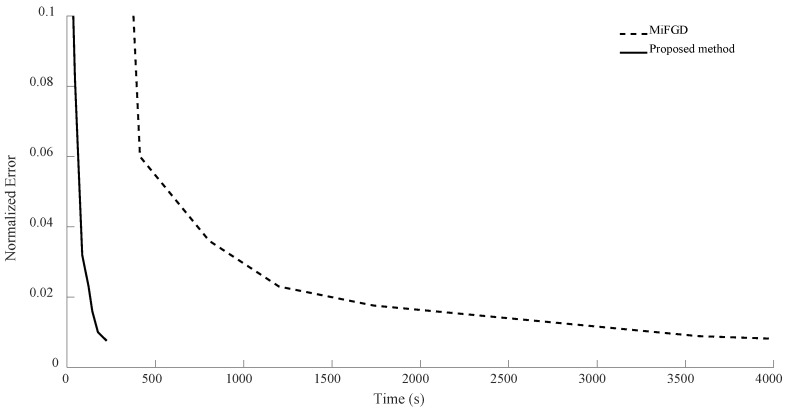
Numerical result for n = 7, $\theta =\frac{\pi }{2}$, and rank = 10.

**Figure 8:**
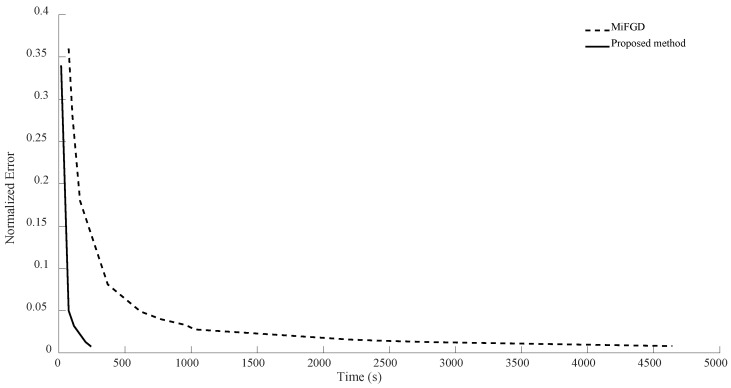
Numerical result for n = 7, $\theta =\frac{\pi }{3}$, and rank = d.

**Figure 9:**
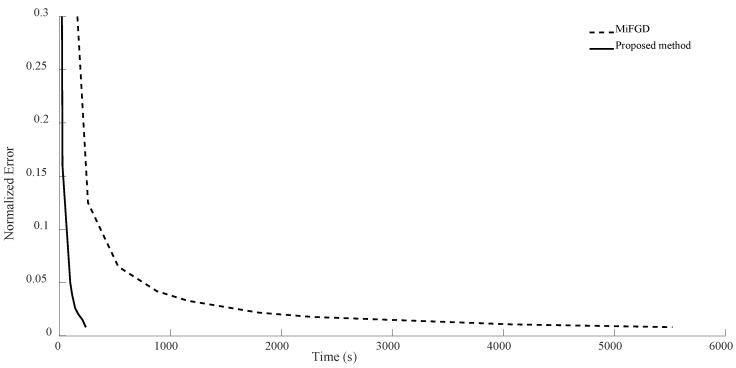
Numerical result for n = 7, $\theta =\frac{\pi }{3}$, and rank = 10.

In the case of n = 7, for the first configuration, MiFGD performed 3229 iterations in 3488 s, which is approximately 1 s per iteration, while the proposed method completed 74 iterations in 225 s, which is 3 s per iteration. However, overall, it performed much better, as can be seen from Figure 6, Figure 7, Figure 8 and Figure 9. Indeed, in the proposed method, in each step, we have to perform three expensive operations to calculate $trace\left(T_{k}T_{k}^{†}M_{i}\right),trace\left(S_{k}S_{k}^{†}M_{i}\right)$ and $trace\left(T_{k}S_{k}^{†}M_{i}\right)$ (as opposed to MiFGD, in which the only expensive operation is the calculation of $trace\left(Z_{k}Z_{k}^{†}M_{i}\right)$), yet the elapsed time decreased. This is because once $trace\left(T_{k}T_{k}^{†}M_{i}\right),\;trace\left(S_{k}S_{k}^{†}M_{i}\right)$ and $trace\left(T_{k}S_{k}^{†}M_{i}\right)$ are computed, due to the optimal choice of η, we have a better value for $trace\left(Z_{k+1}Z_{k+1}^{†}M_{i}\right)$, which can be calculated from the following equation (Equation (23)):

$$trace\left(Z_{k+1}Z_{k+1}^{†}M_{i}\right)=trace\left(T_{k}T_{k}^{†}M_{i}\right)+\eta^{2}trace\left(S_{k}S_{k}^{†}M_{i}\right)+2\eta trace\left(T_{k}S_{k}^{†}M_{i}\right)$$

which approximates the experimental measurement data $y_{i}$ (see Equation (12)).

A summary of the results is presented in Table 3. As can be seen from both the table and the figures, when we restrict the rank of the matrix U to 10, MiFGD struggles more noticeably to converge.

**Figure 10:**
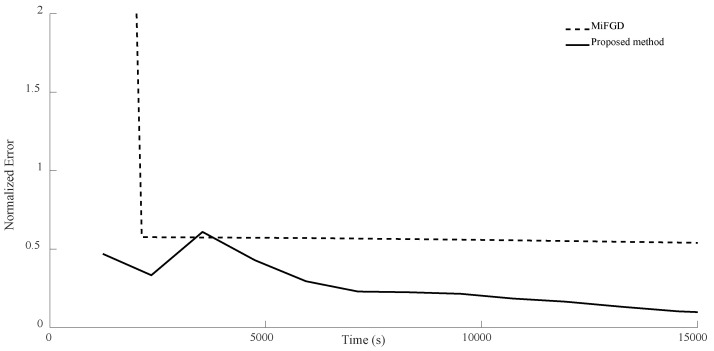
Numerical result for n = 8, $\theta =\frac{\pi }{2}$, and rank = d. Despite initial oscillation, our method outperforms the basic MiFGD.

**Figure 11:**
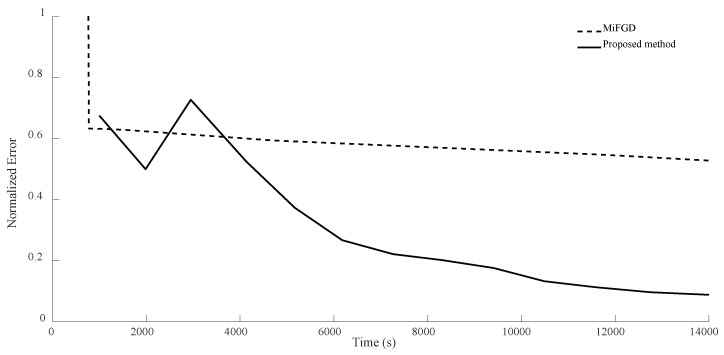
Numerical result for n = 8, $\theta =\frac{\pi }{2}$, and rank = 10. In the first iterations, the proposed method does not seem to be progressing, but as time passes it starts to outperform the basic MiFGD.

**Figure 12:**
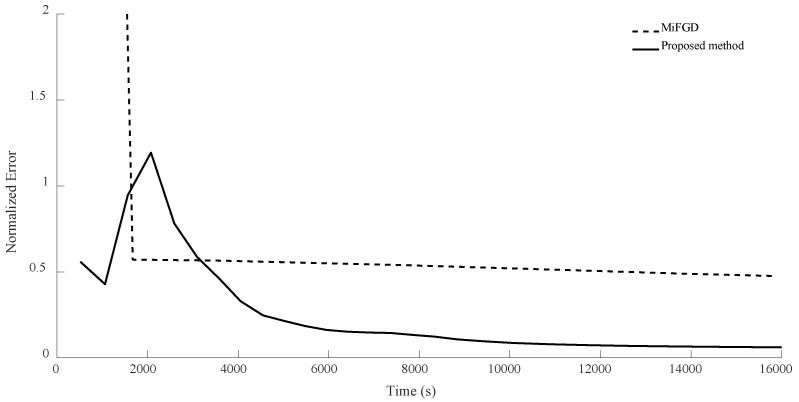
Numerical result for n = 8, $\theta =\frac{\pi }{3}$, and rank = d. Despite initial volatility, the proposed method outperforms the basic MiFGD.

**Figure 13:**
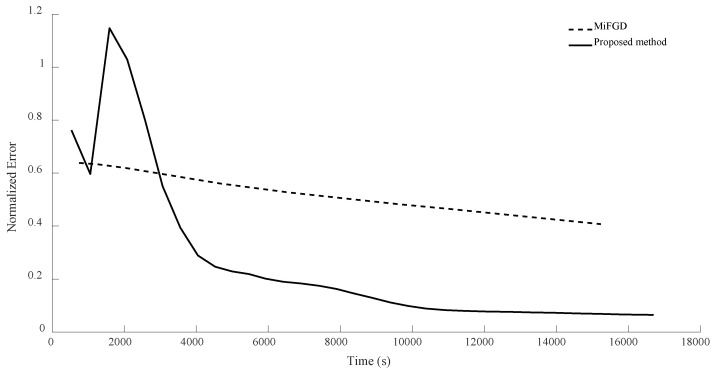
Numerical result for n = 8, $\theta =\frac{\pi }{3}$, and rank = 10. Despite instability at the beginning, the method does a better job compared to the basic MiFGD.

For n = 8, in the first configuration, MiFGD took 617.5 s for each iteration while the proposed method took 1596 s per iteration. This means the proposed algorithm is approximately 2.6 times slower per iteration, as it performs more calculations to avoid an uneducated step, but overall it is much faster to converge, as can be seen from both Table 4 and Figure 10, Figure 11, Figure 12 and Figure 13.

Again, as can be seen from both the table and the figures, when we restrict the rank of the matrix U to 10, MiFGD struggles more noticeably to converge compared to the case in which rank(U) = d.

Another interesting thing is that while the proposed method is supposed to choose the optimal step size, we can see some oscillation noticeably at the beginning. The reason for the oscillation is that, in equations $U_{k+1}=Z_{k}-\eta \left[\sum_{i}\left(trace\left(Z_{k}Z_{k}^{†}M_{i}\right)-y_{i}\right)M_{i}\right]Z_{k}$ and $Z_{k+1}=U_{k+1}+\mu (U_{k+1}-U_{k})$ (Equations (14) and (15)), two different step lengths, η and μ, are involved. What we have undertaken so far in the proposed method is finding the optimal η, while the other one is fixed (μ=0.95). Indeed, the computation of $Z_{k+1}$ will be influenced not only by $U_{k+1}$, but also by $U_{k}$ from the previous steps. Intuitively, μ determines to what extent the method “remembers” the past iterations (for instance, μ=0 means no “memories” from the past iterations). As a result, while the random starting point might be very far from optimal, it gets reflected in the “memory” of the method and it takes a while before it fades away and gets overshadowed by the accumulation of new memories. As a result, we cannot expect a strictly decreasing error. This issue is something that we will address shortly.

In all the previous experiments, we considered μ=0.95. The main reason was that this keeps the number of expensive operations small, but we could have merged (17) and (19) as follows: $Z_{k+1}=\left(1+\mu \right)Z_{k}-{\mu U}_{k}+\eta S_{k}$. This way, by having computed $trace\left(Z_{k}Z_{k}^{†}M_{i}\right),\;trace\left(U_{k}U_{k}^{†}M_{i}\right),trace\left(S_{k}S_{k}^{†}M_{i}\right),\;trace\left(Z_{k}U_{k}^{†}M_{i}\right),\;trace\left(Z_{k}S_{k}^{†}M_{i}\right)$ and $trace\left(U_{k}S_{k}^{†}M_{i}\right)$, and by having a fixed μ, we can quickly (without much calculation) compute $T_{k}=\left(1+\mu \right)Z_{k}-{\mu U}_{k}$ and then compute $trace\left(T_{k}T_{k}^{†}M_{i}\right),\;trace\left(S_{k}S_{k}^{†}M_{i}\right)$ and $trace\left(T_{k}S_{k}^{†}M_{i}\right)$, and then we can calculate the linear combinations of $trace\left(Z_{k}Z_{k}^{†}M_{i}\right),\;trace\left(U_{k}U_{k}^{†}M_{i}\right),\;trace\left(S_{k}S_{k}^{†}M_{i}\right),\;trace\left(Z_{k}U_{k}^{†}M_{i}\right),\;trace\left(Z_{k}S_{k}^{†}M_{i}\right)$ and $trace\left(U_{k}S_{k}^{†}M_{i}\right)$ and plug them into (23) to find the optimal step length η, as before. This means that by increasing the number of expensive operations per iteration from 3 to 6, we will be able to use grid search (for instance, varying μ from 0.01 to 0.99 by increments of 0.01) to adaptively obtain a good choice of μ for every iteration. Below, we compare this new version of adaptively selecting μ versus fixed μ (μ=0.95) for the case of n = 8.

**Figure 14:**
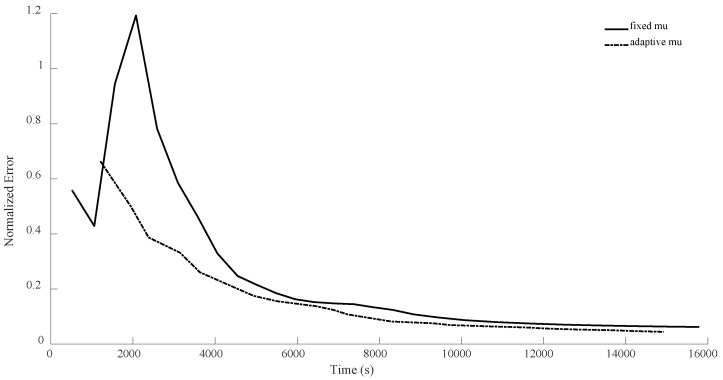
Numerical comparison of the case where μ is fixed versus. the case of adaptively choosing μ for n = 8, $\theta =\frac{\pi }{3}$, and rank = d. As can be seen, the new version does not suffer from instability at the beginning.

**Figure 15:**
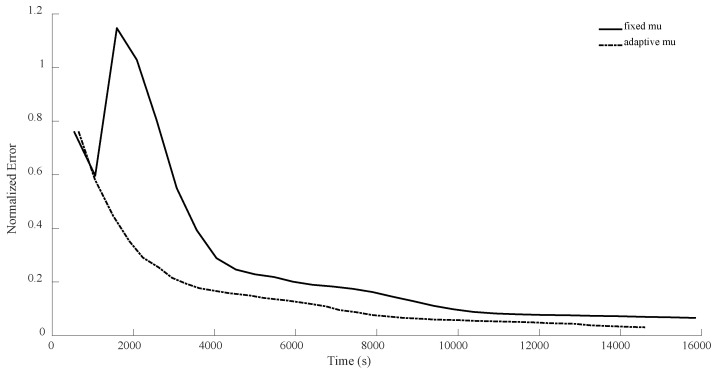
Numerical comparison of the case where μ is fixed versus. the case of adaptively choosing μ for n = 8, $\theta =\frac{\pi }{3}$, and rank = 10. As can be seen, the new version does not oscillate, in contrast to the previous version.

**Figure 16:**
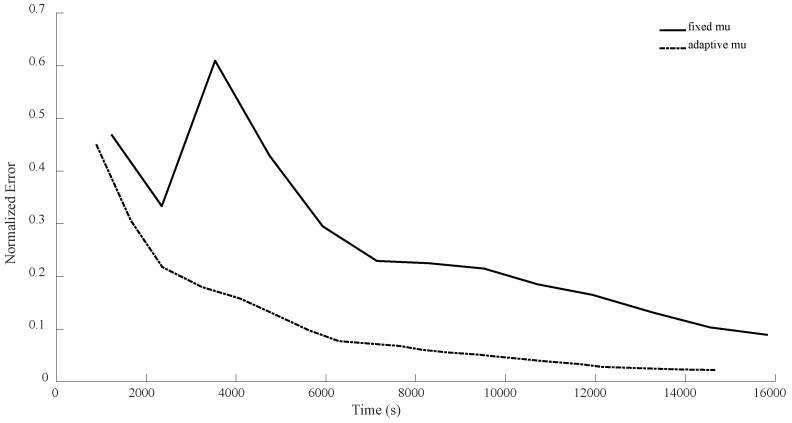
Numerical comparison of the case where μ is fixed versus. the case of adaptively choosing μ for n = 8, $\theta =\frac{\pi }{2}$, and rank = d. As can be seen, the adaptivity of μ helps the new method not to oscillate.

**Figure 17:**
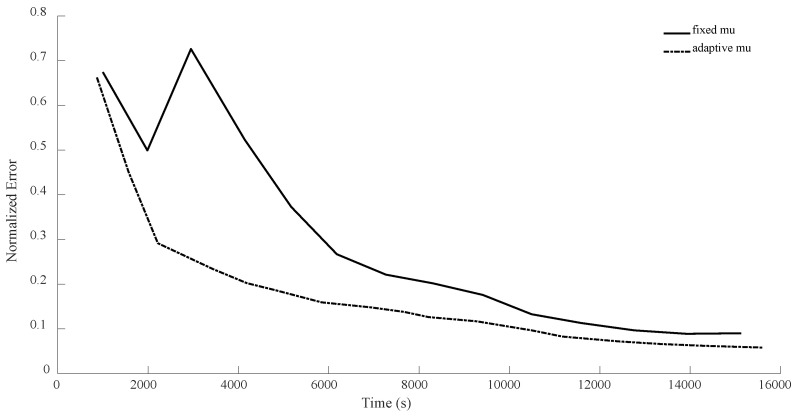
Numerical comparison of the case where μ is fixed versus. the case of adaptively choosing μ for n = 8, $\theta =\frac{\pi }{2}$, and rank = 10. As can be seen, the adaptivity of μ results in a better performance.

As can be seen from the Figure 14, Figure 15, Figure 16 and Figure 17, with this slight modification, we no longer have the oscillatory behavior, and the convergence is slightly faster. Also, note that the computational cost per iteration for the case of adaptive μ is two times the cost for the case of fixed μ, resulting in faster convergence. However, still, it seems that η has the main crucial role compared to μ.

## 5. Conclusions

In this paper, we proposed a novel method for achieving a closed-form solution for optimal step length in an AGD approach to QST, leading to significantly improved performance compared to existing methods. Although the proposed method is three times slower per iteration, the numerical results demonstrate that it is more than ten times faster overall due to the educated step size that is chosen in each iteration. The key innovation of our method lies in the adaptive selection of the step length η, which ensures that each iteration leads to the maximum possible reduction in the objective function. This approach leverages the current state information to make more informed adjustments, thereby enhancing the overall efficiency of the algorithm. Looking ahead, this adaptive step-size strategy opens up several promising avenues for future research. One potential direction is to integrate similar adaptive step-size methods into more sophisticated gradient-based algorithms where the curvature information is better encoded in the iterative approach.

## Figures and Tables

**Table 1:** Comparison of AGD-based methods for QST.

Method	PGDM [2]	MiFGD [13]	Proposed Method
Using SVD	Yes	No	No
AGD	Yes	Yes	Yes
Step size	Fixed	Fixed	Variable
Unit trace	Yes	No	No
Low rank	No	Yes	Yes
# fcn eval	1	1	3

**Table 2:** Comparison of MiFGD and the proposed method for n = 6.

(θ,rank)	MiFGD	Proposed Method
	Time [s], Number of Iterations, Normalized Error	
$\left(\frac{\pi }{2},d\right)$	(318, 3480, 0.0082)	(15, 61, 0.0080)
$\left(\frac{\pi }{2},10\right)$	(364, 3960, 0.0081)	(15, 59, 0.0079)
$\left(\frac{\pi }{3},d\right)$	(497, 5196, 0.0080)	(21, 81, 0.0078)
$\left(\frac{\pi }{3},10\right)$	(578, 6074, 0.0080)	(20, 78, 0.0077)

**Table 3:** Comparison of MiFGD and the proposed method for n = 7.

(θ,rank)	MiFGD	Proposed Method
	Time [s], Normalized Error	
$\left(\frac{\pi }{2},d\right)$	(3488, 0.0081)	(225, 0.0076)
$\left(\frac{\pi }{2},10\right)$	(3973, 0.0082)	(224, 0.0076)
$\left(\frac{\pi }{3},d\right)$	(4640, 0.0082)	(244, 0.0076)
$\left(\frac{\pi }{3},10\right)$	(5523, 0.0082)	(241, 0.0082)

**Table 4:** Comparison of MiFGD and the proposed method for n = 8.

(θ,rank)	MiFGD	Proposed Method
	Time [s], Normalized Error	
$\left(\frac{\pi }{2},d\right)$	(15,439, 0.5384)	(14,550, 0.1032)
$\left(\frac{\pi }{2},10\right)$	(14,396, 0.5239)	(13,915, 0.0890)
$\left(\frac{\pi }{3},d\right)$	(15,782, 0.4764)	(14,992, 0.0630)
$\left(\frac{\pi }{3},10\right)$	(15,236, 0.4073)	(15,213, 0.0685)

## Data Availability

The original contributions presented in the study are included in the article, further inquiries can be directed to the corresponding author.
